# Veterinarians in a Changing Global Climate: Educational Disconnect and a Path Forward

**DOI:** 10.3389/fvets.2020.613620

**Published:** 2020-12-17

**Authors:** Collin G. Kramer, Katherine A. McCaw, Jill Zarestky, Colleen G. Duncan

**Affiliations:** ^1^College of Veterinary Medicine and Biomedical Sciences, Fort Collins, CO, United States; ^2^School of Education, Colorado State University, Fort Collins, CO, United States; ^3^College of Veterinary Medicine and Biomedical Sciences, Department of Microbiology, Immunology and Pathology, Fort Collins, CO, United States

**Keywords:** climate change, education, veterinary medicine, sustainability, environment

## Abstract

**Objective:** To synthesize the beliefs, knowledge and interest of veterinarians on the relationship between veterinary medicine and climate change, with the intent to identify any educational gaps and opportunities.

**Sample:** Responses from 560 U.S., and 54 non-U.S. veterinarians.

**Procedures:** An anonymous, online survey of veterinarians was distributed through electronic media, state and professional associations, and a veterinary magazine advertisement. The survey was conducted between July 1st and December 31st of 2019.

**Results:** Overall, veterinary respondents were confident that climate change is happening, is caused by human activities, and is impacting both human and animal health. Veterinarians also agreed that the profession should have an advocacy role in educating the public on climate change and its health impacts, particularly in clinical practices where environmental sustainability promotion can be shared with clients. Although veterinarians agreed the profession needs to be involved with climate change advocacy, most reported having had no educational opportunities within their veterinary medicine curriculum or access to continuing education on climate change.

**Conclusions and Clinical Relevance:** The results highlight the need for the development of educational opportunities on the topic of climate change such that veterinarians are equipped to address their concerns about current and future animal health threats.

## Introduction

Action is urgently needed to mitigate the profound health threats arising from climate change. Human health professionals around the world have begun to address the issue and leadership is seen at all levels, from international organizations like the World Health Organization (WHO), to local community driven efforts (1, 2). In addition to an emerging field of research on the epidemiology, prevention, and response to climate associated illness, educators are working to integrate climate change into medical curricula (3, 4). These efforts are supported by governing bodies such as the American Medical Association who have enacted policy “aiding physicians in adopting environmentally-sustainable programs in their practices and sharing these concepts with their patients and communities” (5). Simultaneously, there are numerous initiatives underway to reduce the impact of healthcare delivery itself through environmentally sustainable practices in the clinical setting (6).

In contrast, the animal health community has been less enterprising in the area of sustainable animal health and climate change adaptation planning or action. In a recent survey of U.S. veterinary students, respondents highlighted a striking lack of educational opportunities on the topic despite their overwhelming belief that climate change is occurring and has health impacts for both people and animals (7). As doctors of many species with an established role in public and ecosystem health, veterinarians have the potential to meaningfully contribute to climate change action, however education and empowerment are necessary. Failure of veterinarians to engage on such a massive, global health issue has generated concerns for the social relevance of the profession (8).

It is unclear where the divide lies between the animal health community and action on climate change. While veterinary students highlighted educational gaps and concern for political disconnect with clients (7), it is not known if graduate veterinarians share the same perceptions. The objective of this study was to obtain veterinarians' opinions and knowledge about climate change and veterinary medicine, and to determine educational disparities and opportunities surrounding the topic.

## Materials and Methods

The study was based on analogous research projects targeting veterinary students (7) or physician groups (9–11). An anonymous survey of U.S. veterinarians was administered using online survey software^1^. The survey consisted of twenty-three questions including multiple choice, ranking, and multi-select (Appendix 1). Questions were grouped into six categories including demographics, climate change beliefs and knowledge, health-related impacts of climate change, the role of veterinarians in climate change, barriers to veterinarians for addressing climate change, and climate change and health educational opportunities. The survey was reviewed and approved by the Colorado State University Institutional Review Board prior to distribution. The survey link was distributed to veterinarians through social media, state and interest group associations and an advertisement in a veterinary magazine. The survey was open from July 1st to December 31st, 2019. As no question mandated a response in order to complete the survey, data expressed as percentages represent valid percentages to exclude missing data from unanswered questions. The frequency of responses between groups was compared using chi-squared tests. Data analysis was conducted using commercially available software^2^.

## Results

### Demographics of Participants

The survey link was accessed 651 times with 614 respondents answering at least one of the questions. Of the 614 respondents who provided location information, the overwhelming majority (91.2%, 560/614) were from the U.S. and 54 were from 15 other countries. Data was stratified by U.S. and non-U.S. respondents for further analysis. Of the respondents from the U.S., there was at least one respondent from each state except for Hawaii, North and South Dakota (Table 1). Most survey participants were in the 30–39 year age range (35.2%, 184/522), followed by 40–49 year age range (21.1%, 110/522), 50–59 year age range (16%, 83/522), <30 years (13.2%, 69/522), 60–69 year age range (10.7%, 56/522), and >70 years (3.8%, 20/522). The majority of respondents were female (73.9%, 400/541) followed by male (25.5%, 138/541) and (0.6%, 3/541) identifying as other or gender not listed.

**Table 1:** Number of survey participants from each state of the 560 U.S.A. respondents.

**State**	**Participants**	**State**	**Participants**
Arizona	27	Missouri	13
California	31	New York	19
Colorado	104	North Carolina	14
Florida	14	Ohio	12
Georgia	20	Oklahoma	12
Illinois	10	Pennsylvania	10
Iowa	10	Texas	68
Kansas	11	Virginia	19
Maryland	19	Participants from states with <10 responses,	
Minnesota	17	state not reported or multiple states:	149

The majority of U.S. respondents were employed in small animal clinical practice (54.9%, 301/548), followed next by academia (13.5%, 74/548), government (10.4%, 57/548), mixed animal clinical practice (6.9%, 38/548), other (6.8%, 37/548), industry (4.2%, 23/548), and large animal clinical practice (3.3%, 18/548). The most common employment sectors within the “other” category were non-profit shelter medicine (13.5%, 5/37) and specialty medicine (13.5%, 5/37), followed by wildlife (10.8%, 4/37), environmental/public health (8.1%, 3/37), consulting (5.4%, 2/37), equine medicine (5.4%, 2/37), and lab animal (2.7%, 1/37). Most participants were employed in suburban areas (49.6%, 273/550), followed by urban areas (26%, 143/550), rural areas (20%, 110/550), areas other than those listed (4%, 22/550), and those who felt they did not know how to describe the region in which they work (0.4%, 2/550). The most common political belief of the respondents was somewhat liberal (31.8%, 76/553), followed by moderate (24.8%, 137/553), very liberal (19.3%, 107/553), somewhat conservative (15.2%, 84/553), very conservative (7.2%, 40/553), and other (1.6%, 9/553).

### Climate Change Beliefs

**Figure 1:**
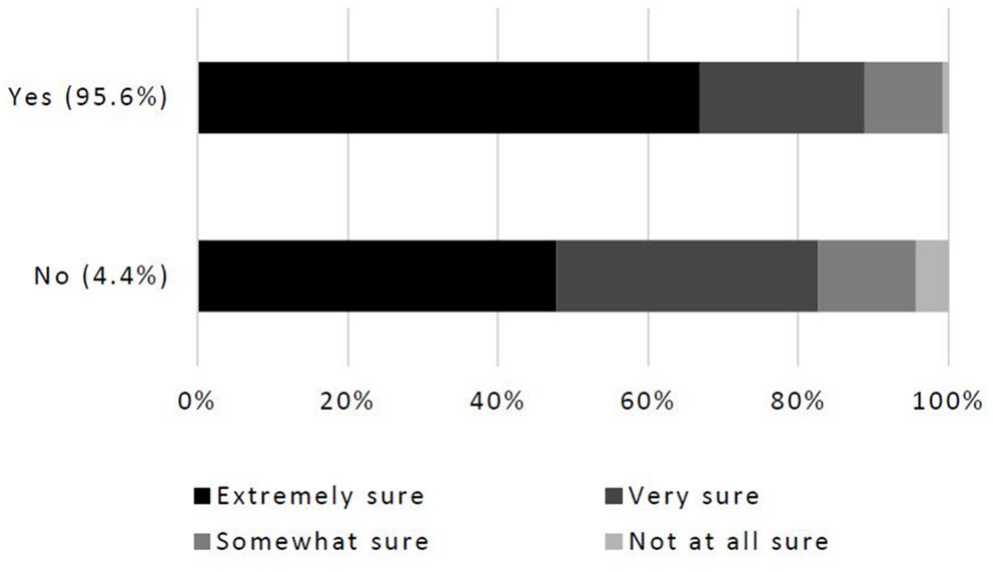
Percentage of veterinarian participants who believe, or do not believe, that climate change is occurring and their level of confidence in that belief (*n* = 521).

The overwhelming majority of U.S. participants (90.8%, 505/556) agreed climate change is occurring at this time. Some participants [4.7% (26/556)] think climate change is not occurring at this time, while 4.5% (25/556) felt they did not know if climate change is occurring. Those who believe climate change is occurring were more confident in their belief than those who do not believe in climate change (Figure 1). Veterinarians feel climate change is caused either mostly (58%, 318/548) or about equally (17%, 93/548) by human activities and natural changes in the environment. Some participants (9.1%, 50/548) think climate change is caused entirely by human activities. Even fewer believe climate change is caused mostly (8.4%, 46/548) or entirely (4%, 22/548) by natural changes in the environment. About 0.5% (3/548) of respondents think climate change is not happening, while 2.9% (16/548) felt they did not know what is causing climate change to occur.

### Climate Change and Health

**Figure 2:**
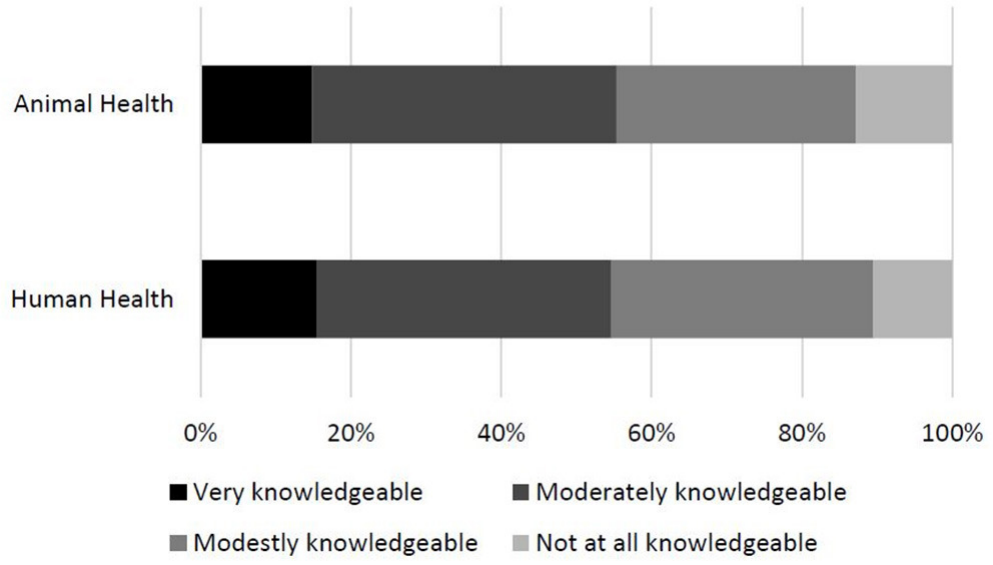
Percentage of veterinarian, self-reported knowledge on the association between climate change and human and animal health (*n* = 544).

Generally, most U.S. veterinarians had some knowledge about the association between climate change and human health impacts with 39.2% (213/548) feeling moderately and 15.4% (84/548) feeling very knowledgeable (Figure 2). Veterinarians reported being similarly knowledgeable about the association between climate change and animal health impacts with 40.4% (220/548) feeling moderately and 14.9% (81/548) feeling very knowledgeable (Figure 2). Over half of the survey participants felt climate change is moderately (43.4%, 238/548) or greatly (18.1%, 99/548) relevant to direct veterinary patient care. While 24.6% (135/548) of veterinarians felt climate change is only a little relevant to direct veterinary patient care, 7.8% (43/548) did not think it was relevant at all, and 6% (33/548) did not know if it was relevant to direct veterinary patient care.

The ways in which veterinarians feel that veterinary patients are expected to be impacted by climate change, both now and in the future, are presented in Table 2. Vector-borne disease, extreme weather and heat-associated illness/stress were issues ranked highest of concern now, while vector-borne disease, extreme weather and declining air quality were the issues ranked the highest of concern for the future. Additional health impacts of concern to respondents included pollution, allergens, loss of medicinal resources found in nature, animal relinquishment or abandonment, loss of biodiversity and general destruction of natural ecosystems. When participants were asked to identify groups of animals they felt would disproportionately experience negative effects from climate change, they recognized rural wildlife (70%, 392/560), production animals (65.9%, 369/560), animals owned or cared for by people with low socioeconomic status (60.4%, 338/560), animals with underlying health conditions (56.8%, 318/560), and urban wildlife (55.9%, 313/560) as the groups they considered to be at highest risk. Less than half of respondents identified older animals (45.4%, 254/560), rural companion (35.4%, 198/560), and urban companion animals (33.8%, 189/560) at increased risk. Another 6.3% (35/560) of participants recognized “other” groups of animals as being at increased risk such as zoo and marine wildlife.

**Table 2:** Ways in which participants believe veterinary patients are currently being affected or will be affected in the next 10–20 years by climate change.

**Time period**	**Yes (%)**	**No (%)**	**Don't know (%)**	**Rank**	***N***
**Increasing vector-borne diseases**					
Now	83.2	6.6	10.2	1	519
Future (10–20y)	86.4	4.2	9.4	1	403
**Increasing extreme weather events**					
Now	80.3	12.9	6.7	2	519
Future (10–20y)	81.3	8.8	9.8	2	407
**Increasing heat-associated illness/stress**					
Now	66.2	19.3	14.5	3	517
Future (10–20y)	73.0	11.5	15.5	5	407
**Increasing water-associated illnesses/stress**					
Now	61.9	17.8	20.3	4	517
Future (10–20y)	75.1	9.9	15.1	4	405
**Declining air quality**					
Now	61.4	20.7	18.0	5	518
Future (10–20y)	75.2	9.6	15.2	3	408
**Reduced food safety, quality and security**					
Now	47.4	32.4	20.2	6	515
Future (10–20y)	60.9	16.4	22.7	6	409

### The Role of Veterinarians

**Figure 3:**
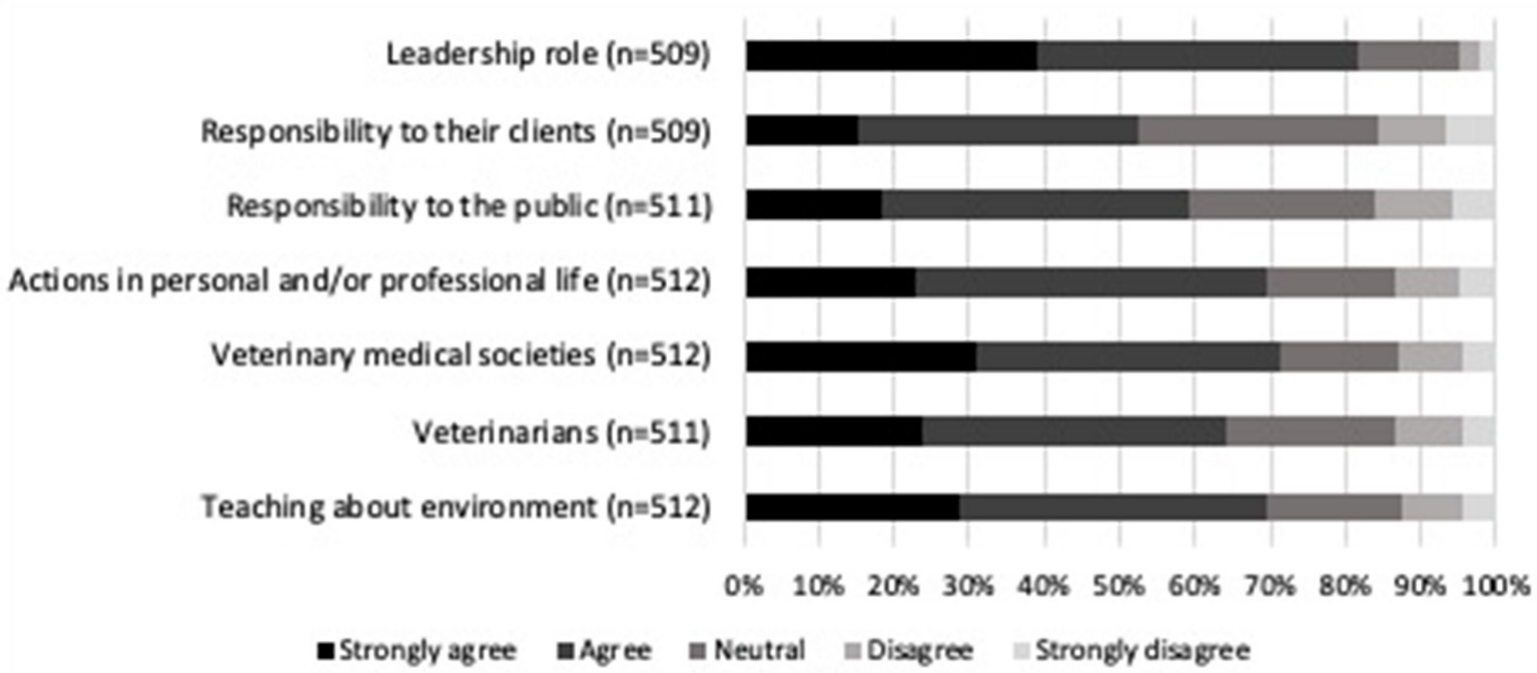
Percentage of level of agreement or disagreement from veterinarians on the potential involvement of the veterinary profession on climate change and health. Complete statements can be found in Appendix 1.

**Figure 4:**
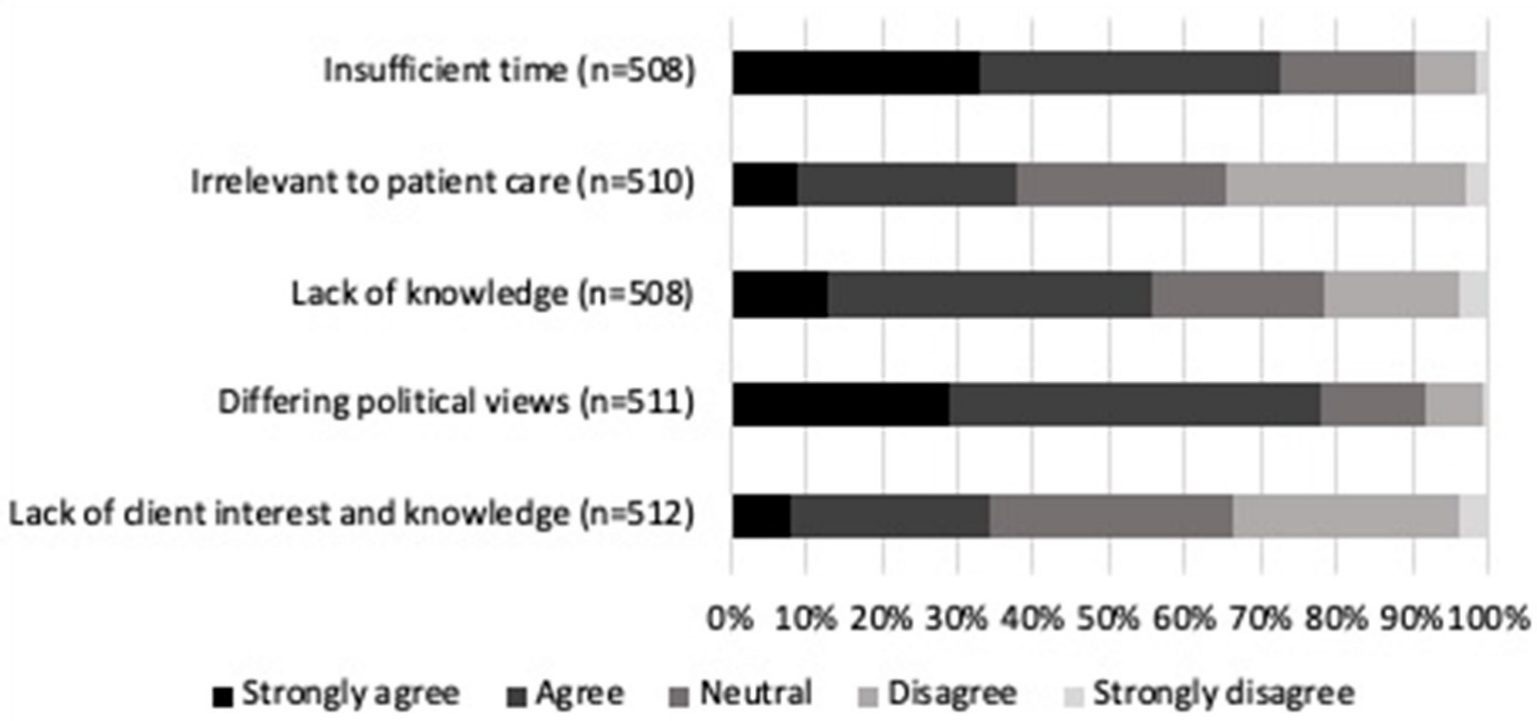
Percentage of level of agreement or disagreement from veterinarians on potential barriers preventing veterinarians from addressing climate change-related health issues with clients. Complete statements can be found in Appendix 1.

Regarding participants' opinions of the role veterinarians play concerning climate change and health (Figure 3), there was strong agreement (81.7%, 416/509) that veterinarians should have a leadership role in encouraging environmental sustainability amongst offices, clinics, and hospitals. Respondents felt veterinary medical societies should have a significant advocacy role in climate change and health (71.5%, 366/512). Similarly, participants agreed (69.7%, 357/512) that the actions they take in their personal and/or professional lives can contribute to effective action on climate change. They also agreed that teaching about the environment and its association with health impacts should be incorporated into veterinary medical education (69.7%, 357/512). The least agreed upon statements, while still supported by more than half of the participants, were regarding the responsibility of veterinarians to bring health effects of climate change to the attention of the public (59.3%, 303/511) and their clients (52.7%, 268/509). Potential barriers that participants felt were likely to prevent veterinarians from addressing climate change-related health issues with clients can be found in Figure 4. Differences in political views damaging the veterinarian-client-patient relationship after discussing climate change was identified as the largest barrier (77.7%, 397/511). Some respondents felt that addressing these issues with veterinary clients will not make much difference in their patients' overall health (37.8%, 193/510).

### Educational Opportunities on Climate Change

When U.S. participants were asked about climate change educational opportunities provided during their training by the respective veterinary programs, 86.7% (437/504) stated that no educational opportunities were available to them. Only 10.1% (51/504) of participants reported that educational opportunities did exist for them in the form of clubs (such as One Health, chapter of the Association for Veterinarians for the Environment, Public Health) or through core and elective courses [like Epidemiology, Parasitology, Public Health, Population Medicine, and One Health). Some participants (3.2% (16/504)] were unaware of any education opportunities during their training. Of the available mechanisms through which climate change education could be provided to practicing veterinarians, continued education such as conferences and seminars was the most commonly selected option (70.7%, 396/560), followed by core content in the veterinary medical curriculum (42%, 235/560) and elective opportunities through veterinary societies (40%, 224/560). A small number of participants felt they did not know (4%, 22/560). The most commonly shared write-in response was journal articles (6.5%, 2/31).

**Figure 5:**
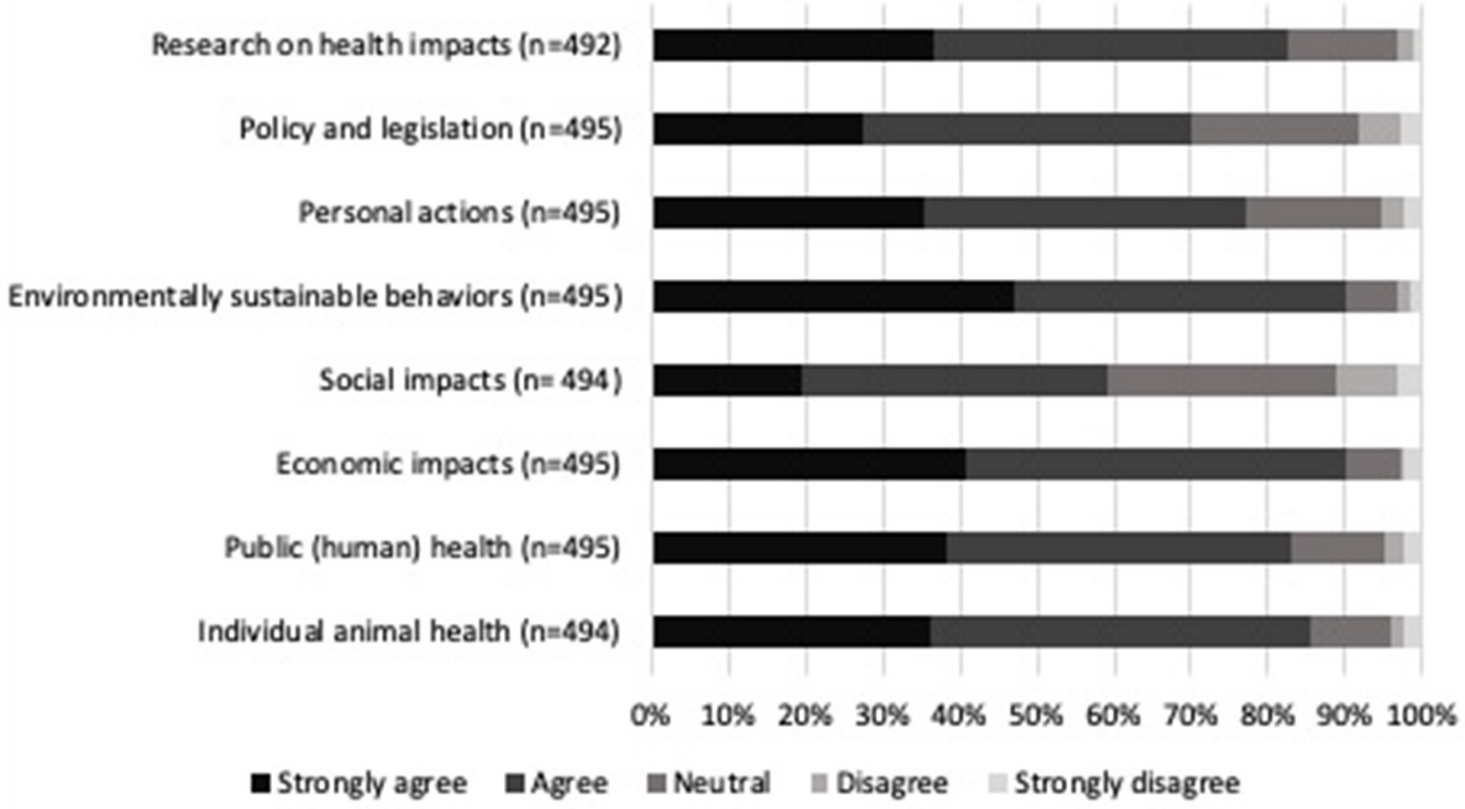
Percentage of level of agreement or disagreement from veterinarians on climate change and health topics that the veterinary community should be knowledgeable about. Complete statements can be found in Appendix 1.

**Figure 6:**
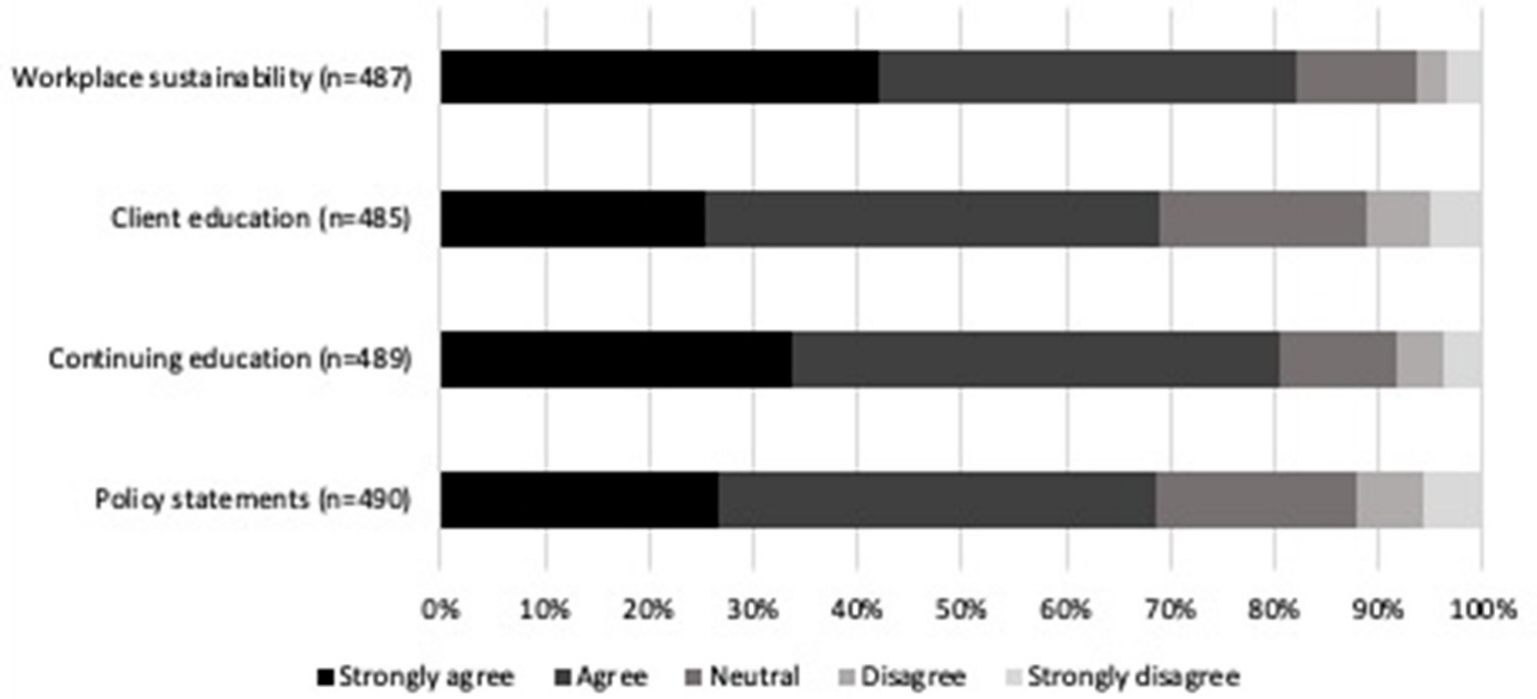
Percentage of level of agreement or disagreement from veterinarians on helpful climate change and health-related resources that they may find helpful to use in practice. Complete statements can be found in Appendix 1.

Climate change topics that participants felt the veterinary community should be knowledgeable about can be found in Figure 5. The topics respondents chose as the highest priority for the veterinary community to be knowledgeable about were the economic impacts of climate change as related to animals (90.1%, 446/495) and environmentally sustainable behaviors specific to veterinary medical practice (89.9%, 445/495). Interest in a variety of climate and health resources is presented in Figure 6. Participants were most interested in receiving guidance on how to make their workplaces sustainable (82.1%, 400/487) and having access to continuing education opportunities on climate change and health (80.4%, 393/489). The most common open text resource suggestion was access to journal articles and current research on climate change. Other suggestions were continuing education and online resources, community outreach, policy and advocacy, methods to reduce biomedical waste, and access to environmentally friendly supplies.

In addition to the open text responses specific to questions described above, numerous respondents made comments pertaining to the impacts of climate change on veterinarian well-being. These included feeling blamed for animal contributions to climate change (ex. methane), exasperated financial stress secondary to economic ramifications, direct human health effects, and an inability to take action on climate change given the already overwhelming pressures of the profession. Respondents also reported the mental health impacts climate change imposes on veterinarians due to the concerns for animals and the environmental determinants of health upon which they are dependent.

### Non-U.S. Responses

Although the survey targeted veterinarians from the U.S., 54 veterinarians from outside the U.S. also took the survey and responses were compared to those of the U.S. This group represented fifteen different countries of which Canada was most strongly represented (*n* = 18). The non-U.S. respondents did not differ from the U.S. respondents in gender or age (*p* > 0.05 for both); however, they were generally more non-clinical (academic, industry or government) than clinical (all practice types, *p* = 0.02) and more liberal (*p* = 0.01). All non-U.S. participants felt that climate change is happening at this time and more of them believe it is caused by humans (*p* = 0.02). Their level of knowledge regarding the human or animal health impacts of climate change does not differ from U.S. veterinarians (*p* > 0.05) however they more strongly believe that climate change is directly relevant to patient care (*p* = 0.02). In general, non-U.S. participants agreed with U.S. veterinarians with respect to how (*p* > 0.05 for all), and which, animals are impacted by climate change except that statistically more non-U.S. veterinarians were concerned about extreme weather and heat (*p* < 0.05).

When asked about potential barriers that would prevent talking about climate change with clients, non-U.S. respondents were significantly less concerned about different political views of their clients (*p* = 0.02) and had a significantly stronger belief that they could make a difference in animal health (*p* < 0.01). They did share the concerns of U.S. veterinarians regarding time in appointments, client interest and lack of knowledge on the topic (*p* > 0.5 for all). Significantly more non-U.S. respondents felt that teaching about the environment and its association with health impacts should be incorporated into veterinary medical education, that veterinarians and veterinary medical societies should have a significant advocacy role in relation to climate change and health, and that veterinarians have a responsibility to bring the health effects of climate change to the attention of the public and their clients (*p* < 0.05 for all). Non-U.S. veterinarians were similar to U.S. veterinarians in their belief that veterinarians should have a leadership role in encouraging offices, clinics, and hospitals to be as environmentally sustainable as possible and that actions they take in their personal and professional lives contribute to effective action on climate change. As with U.S. respondents, the overwhelming majority of non-U.S. veterinarians (89.5%, 43/48, *p* > 0.05) did not learn, or do not remember learning, about climate change as part of their education. There was no statistically significant difference (*p* > 0.05 for all) between U.S. and non-U.S. veterinarians regarding the topics that veterinarians should be knowledgeable about and the mechanisms in which it could be delivered.

## Discussion

Veterinary respondents overwhelmingly agree that climate change is occurring and that it is associated with a variety of human and animal health impacts. Overall, these results are similar to those of veterinary students (7) and human physicians (9–11). The majority of the respondents, and the target population, were U.S. veterinarians with a gender and employment distribution similar to the national average (12). Participants reported working in all types of communities (urban, suburban, rural) and shared diverse political beliefs spanning the political range.

Collectively, results of this study strongly support the need for veterinary education on the health impacts of climate change. Ninety percent of respondents had no, or were not aware of, exposure to this topic during their veterinary school education. This is in contrast to current veterinary students where 76% of respondents had no, or were not aware of, educational opportunities within their curriculum (7), indicating only a modest shift in veterinary training over time despite increased demand. Consistent with students, veterinary respondents reported being equally knowledgeable about the health impacts of climate change in humans as in animals, which is surprising given the focus of veterinary education is on animals. This result suggests that some of respondents' learning about climate change has been obtained from sources of information unrelated to or separate from their work with animals.

Most veterinarians recorded that they believe education on climate change should be provided through continuing education (CE) opportunities while others felt that incorporating educational opportunities into the core content of veterinary curricula would be the best option. Some veterinary schools have already integrated climate change into their curricula through classes such as preventative medicine, epidemiology, population medicine, and parasitology, indicating that it may not be as difficult as predicted to incorporate the topic of climate change into the core content. Another potential avenue for incorporating climate change education into the core curricula emphasizes building on existing models (i.e., One Health, epidemiology or preventative medicine classes, etc.) to reinforce intersecting health concepts (13). However, some respondents noted that veterinary curricula are already saturated with content and adding more coursework could be overwhelming.

A focus on CE may be a more feasible solution; creating such opportunities at conferences or through professional associations such as the AVMA, could be well-received. Such efforts could focus on priorities indicated by this, and previous (7), studies' respondents, including “encouraging offices, clinics, and hospitals to be as environmentally sustainable as possible.” Veterinarians also prioritized knowledge about the economic impacts of climate change as related to animals. As every veterinary school in the United States has a Veterinary Business Management Association (VBMA) chapter with a business certificate program (14), the economic impacts of climate change on animal health could be integrated as a critical part of a CE program.

CE that addresses other challenges, such as difficult conversations with clients or colleagues around climate change, may appeal to others. Respondents felt that differences in political views damaging the veterinarian-client-patient relationship coupled with the lack of time during appointments are the largest potential barriers to addressing climate change-related health impacts with clients. Although the concern that political opinions and differences of beliefs on climate change would inhibit progressive conversation, studies have shown that people, regardless of political party affiliation, are positively responsive to discussing climate change when taken from an approach based on health impacts (15–18). Development of CE programs that consider varying political orientations will require collaboration between health professionals and environmental education researchers (17). CE oriented on communications and difficult conversations for veterinarians to instruct them on the proper and least confrontational verbiage to use with clients could also be a path forward.

The veterinary profession, like other disciplines, could start providing continuing education opportunities on climate change through conventions and webinar series (19). Online learning has the potential to reach a broad audience, is accessible to many who do not have the time or resources to travel to in-person trainings and may exist in convenient online venues that people already visit (e.g., Facebook and LinkedIn). Certificate programs or other micro-credentials may provide useful motivation for individuals or practices to complete a training. In a recent survey of pet owners, most respondents valued knowing that their veterinary clinic had obtained a certification for their sustainability practices (20) suggesting that such educational programs may have economic co-benefits.

Despite the excellent learning opportunities that exist in many spheres, individual-level learning about conservation and sustainability does not typically translate to behavior change without clear motivational and social supports (21). Theories of social practice suggest collective behavior change is constrained by culture and context (22). Learning and subsequent action needs to be clearly connected to issues of local relevance (23), and supported by behavioral norms (24). In other words, it is easier to change people's behaviors when they are working to address a concrete, local issue rather than an abstract or distant one, and if the desired behaviors are socially acceptable.

With the social and contextual constraints in mind, approaches to behavior change need to provide more support for veterinarians who wish to learn more about climate change, build the sustainability efforts of their practices, and influence those around them, including clients, colleagues, and loved ones. Approaches such as Communities of Practice (25) are intended to provide support for participants' shared learning and may help mitigate negative messages learners receive elsewhere. Voluntary education grounded in social interaction has the downside that, at first, such efforts are likely to attract only like-minded individuals, meaning that skeptics are unlikely to participate. Yet as a community grows, it may start to shift the larger professional culture and, in this case, put useful social pressure on skeptics.

Building community around a shared area of interest may have additional co-benefits to veterinarians. Despite not being a topic on the survey, numerous write-in responses highlighted the impact of climate change on veterinarians' mental health and well-being. The direct and indirect mental health impacts associated with climate change are vast and increasing (26, 27). Veterinarians already struggle with significant mental health issues (28, 29); however, finding meaningful purpose, relationships, and personal growth can promote professional well-being (30). By supporting concerned veterinarians to engage in collective action on climate change, we may save more than just our environment.

Veterinary respondents agreed that their professional organizations could contribute through the development of policy statements on the topic and these are a logical place to begin building community. While this type of leadership has been seen in human medicine (5), it appears inconsistent in the animal health sector. As an example, when entering the profession, veterinarians around the world take a public oath of professionalism and standards. This declaration has evolved to reflect societal concerns such as animal welfare and environmental stewardship, however there are notable differences between countries (31). While the World Veterinary Association includes “to advocate for the sustainable use of terrestrial, aerial, and aquatic animals in their diverse ecosystems through stewardship to reduce environmental impacts,” (32) and an interprofessional planetary health pledge has been proposed (33), there is no reference to the environment in the Veterinarians Oath from the AVMA (34). Although this omission has been postulated to reflect a national sentiment (31), it does not appear to reflect the perceptions of veterinarians responding to this survey as more than 90% believe that climate change is occurring, a number significantly higher than the 73% of the American general public (35). It may be appropriate for veterinary medical associations to assess their policies to ensure they best support their constituents and the public.

While the survey was designed for U.S. veterinarians, responses from non-U.S. veterinarians provided an interesting comparison group. Veterinarians outside of the U.S. were generally less concerned about politics, more strongly believed that climate change is directly relevant to patient care and feel more empowered to act beyond their personal and professional behaviors. While this small number of non-U.S. responses is grossly insufficient to make strong conclusions, it highlights areas for additional research. Similarly, given the convenience sampling of U.S. veterinarians, further work is needed to determine if the results of the present study are generalizable beyond the response group.

Climate change is the biggest global health threat of the Twenty-first century (1) and therefore of direct relevance to health professionals. Veterinarians, current and future (7), are similarly concerned about this growing health threat, but have received minimal to no formal education on the topic. However, taking action on climate change is within the scope of veterinary practice (8). Through the development of climate change educational opportunities and fostering a sense of community around these ideals, we can support veterinarians to be influential stewards of human, animal and environmental health.

## Data Availability Statement

The original contributions presented in the study are included in the article/Supplementary Material, further inquiries can be directed to the corresponding author.

## Ethics Statement

The survey was reviewed and approved by the Colorado State University Institutional Review Board prior to distribution. Participants provided their informed consent to participate in this study.

## Author Contributions

All authors were part of the design, execution and analysis of the project and were involved in drafting and approving the final manuscript.

## Conflict of Interest

The authors declare that the research was conducted in the absence of any commercial or financial relationships that could be construed as a potential conflict of interest.

## Footnotes

^1^Qualtrics^®^, Provo, UT.

^2^SPSS Statistics v25, IBM Inc., Armonk, NY.
