# Supplementary material for: Veterinarians in a Changing Global Climate: Educational Disconnect and a Path Forward
Source: Front Vet Sci. 2020 Dec 17;7:613620. doi: 10.3389/fvets.2020.613620 (PMC7773640; doi:10.3389/fvets.2020.613620)
Supplement: Supplementary file 1 [file Data_Sheet_1.docx]

**Appendix: Survey questions**

**Q1** Are you a veterinarian?

- Yes
- No

**Q2** In which state do you practice?

- Alaska
- Arizona
- Arkansas
- California
- Colorado
- Connecticut
- Delaware
- Florida
- Georgia
- Hawaii
- Idaho
- Illinois
- Indiana
- Iowa
- Kansas
- Kentucky
- Louisiana
- Maine
- Maryland
- Massachusetts
- Michigan
- Minnesota
- Mississippi
- Missouri
- Montana
- Nebraska
- Nevada
- New Hampshire
- New Jersey
- New Mexico
- New York
- North Carolina
- North Dakota
- Ohio
- Oklahoma
- Oregon
- Pennsylvania
- Rhode Island
- South Carolina
- South Dakota
- Tennessee
- Texas
- Utah
- Vermont
- Virginia
- Washington
- West Virginia
- Wisconsin
- Wyoming
- Other (please list) _________

**Q3** How old are you?

- <30 years
- 30-39 years
- 40-49 years
- 50-59 years
- 60-69 years
- >70 years

**Q4** With which gender do you identify?

- Male
- Female
- Gender not listed

**Q5** What area of veterinary medicine best describes the majority of your veterinary career?

- Small animal clinical practice
- Large animal clinical practice
- Mixed animal clinical practice
- Academia
- Industry
- Government
- Other, please list ______________

**Q6** Which of the following best describes the region in which you work?

- Urban area
- Suburban area
- Rural area
- Other, please list __________________
- I don’t know

**Q7** In general, do you think of yourself as:

- Very liberal
- Somewhat liberal
- Moderate, middle of the road
- Somewhat conservative
- Very conservative
- Other

**Q8** Climate change refers to the idea that the world’s average temperature has been increasing over the past 150 years, may be increasing more in the future, and that the world’s climate is changing as a result. Do you think that climate change is happening at this time?

- Yes
- No
- I don't know

*If ‘Yes’ or ‘No’ was selected for Q8: Skip to Q9*

*If ‘I don’t know’ was selected for Q8: Skip to Q10*

**Q9** How sure do you feel about your previous answer (if climate change is happening)?

- Extremely sure
- Very sure
- Somewhat sure
- Not at all sure

**Q10** Do you think climate change over the past 150 years was:

- Caused entirely by human activities
- Caused mostly by human activities
- Caused about equally by human activities and natural changes in the environment
- Caused mostly by natural changes in the environment
- Caused entirely by natural changes in the environment
- None of the above, because climate change is not happening
- I don't know

**Q11** How knowledgeable do you feel about the association between climate change and **human** health impacts?

- Very knowledgeable
- Moderately knowledgeable
- Modestly knowledgeable
- Not at all knowledgeable
- Not applicable because climate change is not happening

**Q12** How knowledgeable do you feel about the association between climate change and animal health impacts?

- Very knowledgeable
- Moderately knowledgeable
- Modestly knowledgeable
- Not at all knowledgeable
- Not applicable because climate change is not happening

**Q13** How much, if at all, do you think climate change is relevant to direct veterinary patient care?

- A great deal
- A moderate amount
- Only a little
- Not at all
- I don’t know

**Q14/Q15** In which of the following ways, if any, do you think veterinary patients are currently being affected by climate change or might be affected in the next 10-20 years?

|  | Currently | | | In the next 10-20 years | | | |  |
| --- | --- | --- | --- | --- | --- | --- | --- | --- |
|  | Yes | No | Unsure | | Yes | No | Unsure | |
| Declining air quality |  |  |  | |  |  |  | |
| Increasing extreme weather events |  |  |  | |  |  |  | |
| Increasing vector-borne diseases |  |  |  | |  |  |  | |
| Increasing water-associated illnesses/stress |  |  |  | |  |  |  | |
| Reduced food safety, quality and security |  |  |  | |  |  |  | |
| Increasing heat associated illness/stress |  |  |  | |  |  |  | |
| Other, please list |  |  |  | |  |  |  | |
| Other, please list |  |  |  | |  |  |  | |

**Q16** Which, if any, of the following groups will disproportionately experience negative health effects from climate change? [check all that apply]

- Urban companion animals
- Rural companion animals
- Production animals (ex. food, fiber, other)
- Urban wildlife
- Rural wildlife
- Older animals
- Animals with underlying health conditions
- Animals owned or cared for by individuals with low socioeconomic status
- Other, please list ________________________________________________

**Q17** The statements below are intended to gauge your assessment of possible barriers which would prevent you from discussing climate change-related topics with your clients. Please rate the following:

|  | Strongly Agree | Agree | Neutral | Disagree | Strongly Disagree |
| --- | --- | --- | --- | --- | --- |
| Veterinary clients would not be interested or knowledgeable enough to discuss this issue |  |  |  |  |  |
| Differences in political views may damage the veterinarian-client-patient relationship if I brought this issue up in the exam room |  |  |  |  |  |
| Lack of knowledge regarding how to approach the issue with my clients |  |  |  |  |  |
| Addressing these issues with veterinary clients will not make much difference in their animal’s overall health |  |  |  |  |  |
| There is not enough time in a regular client appointment to discuss these issues |  |  |  |  |  |
| Other, please specify: |  |  |  |  |  |
| Other, please specify: |  |  |  |  |  |

**Q18** How much do you agree or disagree with the following statements?

|  | Strongly Agree | Agree | Neutral | Disagree | Strongly Disagree |
| --- | --- | --- | --- | --- | --- |
| Teaching about environment (e.g., climate change) and its association with health impacts should be integrated into veterinary medical education |  |  |  |  |  |
| Veterinarians should have a significant advocacy role in relation to climate change and health |  |  |  |  |  |
| Veterinary medical societies should have a significant advocacy role in relation to climate change and health |  |  |  |  |  |
| I feel that actions I take in my personal and/or professional life can contribute to effective action on climate change |  |  |  |  |  |
| Veterinarians have a responsibility to bring the health effects of climate change to the attention of the public |  |  |  |  |  |
| Veterinarians have a responsibility to bring the health effects of climate change to the attention of their clients |  |  |  |  |  |
| Veterinarians should have a leadership role in encouraging offices, clinics, hospitals to be as environmentally sustainable as possible |  |  |  |  |  |

**Q19** Through what mechanism do you believe education on health impacts from climate change should be provided? [select all that apply]

- Core content in the veterinary medical curriculum
- Elective content in the veterinary medical curriculum
- Elective opportunities through veterinary societies
- Continuing education opportunities (ex. conferences, seminars)
- Other, please specify ________________________________________________
- I don’t know

**Q20** Within your veterinary medical program, did you learn about health impacts associated with climate change?

- Yes, if so please explain (e.g. clubs, elective rotations, elective classes) ___________________________
- No
- I don't know

**Q21** Which of the following climate change and health topics do you believe the veterinary community should be knowledgeable about?  Please select your level of agreement with the statements below.

|  | Strongly Agree | Agree | Neutral | Disagree | Strongly Disagree |
| --- | --- | --- | --- | --- | --- |
| Individual animal health impacts of climate change |  |  |  |  |  |
| Public (human) health impacts of climate change |  |  |  |  |  |
| Economic impacts of climate change as related to animals (example: production animals) |  |  |  |  |  |
| Social impacts of climate change |  |  |  |  |  |
| Environmentally sustainable behaviors specific to veterinary medical practice (ex. biomedical waste, building design) |  |  |  |  |  |
| Personal actions to reduce environmental footprint (e.g. transportation, food choices, energy use, water use) |  |  |  |  |  |
| Policy and legislation relevant to climate change and health |  |  |  |  |  |
| Research on the health impacts of climate change |  |  |  |  |  |
| Other, please explain |  |  |  |  |  |

**Q22** How helpful would climate & health related resources be to you as a veterinarian?  Please select your level of agreement with the following options.

|  | Strongly Agree | Agree | Neutral | Disagree | Strongly Disagree |
| --- | --- | --- | --- | --- | --- |
| Policy statements provided by my professional associations |  |  |  |  |  |
| Continuing education on climate change and health |  |  |  |  |  |
| Client education materials |  |  |  |  |  |
| Guidance on how to make my workplace sustainable |  |  |  |  |  |

**Q23** What other resources, if any, would be helpful to you as a veterinarian? ___________________
